# Assessment of the neutrophil-to-lymphocyte ratio as a prognostic marker in patients with newly diagnosed diffuse large B-cell lymphoma: A Colombian Cohort Study

**DOI:** 10.1016/j.htct.2025.106237

**Published:** 2025-12-12

**Authors:** Paula María Sánchez, Juan Felipe Combariza-Vallejo

**Affiliations:** Hematology Department, Clínica Universitaria Colombia. Clínica Colsanitas S.A. Bogotá, Colombia

**Keywords:** Diffuse large B-cell lymphoma, Prognosis, Survival, Neutrophil-to-lymphocyte ratio (NLR)

## Abstract

**Introduction:**

Diffuse large B-cell lymphoma is a complex disease, and prognostic scores are inadequate for identifying high-risk patients. Recently, leukocyte indices, like the neutrophil-to-lymphocyte ratio, have become a marker of prognosis. The purpose of this study is to evaluate the performance of this marker as a risk predictor in adult patients with newly diagnosed diffuse large B-cell lymphoma in Colombia.

**Materials and methods:**

A retrospective cohort study, calculated the neutrophil-to-lymphocyte ratio and its performance as a predictor for 2-year progression-free survival. Patients were divided into two groups; patients with high ratios in Group One and patients with low ratios in Group Two. Both groups were followed for at least 24 months from diagnosis.

**Results:**

The cohort comprised 198 patients with a median age at diagnosis of 61 years. A neutrophil-to-lymphocyte ratio cutoff point of 6.2 was calculated. Patients with ratios higher than 6.2 (*n* = 45) were placed in Group One, and the patients with ratios below 6.2 (*n* = 153) in Group Two. The median follow-up time was 45 months. The 24-month progression-free survivals were 55.2 % (95 % confidence interval: 42.3–71.9 %) and 73.2 % (95 % confidence interval: 66.2–81.0 %) for high and low ratios, respectively (Hazard ratio 0.62; 95 % confidence interval: 0.44–0.89; p-value = 0.009). The 24-month overall survivals were 70.7 %; (95 % confidence interval: 58.5 - 85.5 %) and 80.4 %; (95 % confidence interval: 66.2–87.3 %), respectively.

**Conclusion:**

A neutrophil-to-lymphocyte ratio with a cutoff point at ≥6.2 could differentiate a diffuse large B-cell lymphoma population with an unfavorable prognosis for progression-free survival.

## Introduction

The most prevalent non-Hodgkin's lymphoma (NHL) in Colombia is Diffuse Large B-Cell Lymphoma (DLBCL), which accounts for 40 % of all cases [1]. Immuno-chemotherapy with the R-CHOP (rituximab, cyclophosphamide, doxorubicin, prednisone, and vincristine) regimen is the standard first-line treatment with remission rates between 60 % and 70 % and overall survival (OS) rates of 85–88 % [2, 3, 4]. About 30 % of the patients experience relapse, which may be caused by the biology of the tumor [5]. Knowledge of the tumor microenvironment could give clues about the uncontrolled growth of abnormal clones, resistance and refractoriness to treatment with chemo-immunotherapy [6].

Various criteria are currently employed in clinical practice for prognostication. Based on this stratification, efforts have been made to establish intensified chemotherapy protocols for patients classified as having intermediate risk (IR) or high risk (HR), given their increased susceptibility to relapse. Despite these efforts, the OS of patients has not improved [6, 7, 8]. Intensive chemotherapy regimens for HR patients have not improved progression-free survival (PFS) rates assessed using the International Prognostic Index (IPI) [9].

Different prognostic factors besides the traditional ones could improve the classification of patients with DLBCL and optimize treatment. The neutrophil-to-lymphocyte ratio (NLR) is proposed as a prognostic marker. The growth of tumor cells is sustained through interaction with surrounding cells; the tumor clone produces cytokines such as CXCL-10, which increase and activate inflammatory cells like macrophages, neutrophils, and dendritic cells, resulting in tumor proliferation, cell migration, neutrophil infiltration, and lymphocyte inhibition [8,10,11]. Elevated neutrophil levels are associated with angiogenesis induction, tumor necrosis factor (TNF) secretion, nitric oxide production, neutrophil trap formation, apoptosis inhibition, and DNA damage [12, 13, 14]. Low lymphocyte counts impair host immunity, leading to decreased recognition and destruction of tumor cells [4,15,16]. NLR balances inflammation pathway activity and autoimmune function [17].

The inflammation and immune changes caused by the tumor are reflected in scores based on peripheral blood count ratios. Additional information about prognosis could be gained through the presence of systemic inflammatory responses that are caused by tumorigenesis [18]. A high NLR has been reported as a factor for poor prognosis in DLBCL with decreased OS and PFS. However, the majority of studies reported were carried out in Asian populations and a wide range of cutoff values were reported (between 2.32 and 5.54) [16,17,19, 20, 21]. In one study of Latin American patients, the NLR was shown to be an adverse prognostic factor with a cutoff value >4 [8], but another study of Peruvian patients identified a cutoff value >6 [22].

The Latin American population is heterogeneous, and the distribution of NHL subtypes varies significantly by geographic region. The DLBCL subtype of lymphomas is more common in Brazil, Guatemala, Peru and Colombia than in Argentina and Chile [1,23]. It is possible that DLBCL patients from tropical zones of Latin American, and Caribbean countries have outcomes that are not the same as those from other regions. This study aimed to evaluate the behavior of the NLR regarding PFS in patients with DLBCL in a Colombian cohort.

## Materials and methods

### Study type

This is a retrospective analytical cohort study. The purpose of the study was to analyze the NLR as an indicator of prognosis in newly diagnosed DLBCL patients and its effect on the PFS and OS.

### Inclusion criteria

Patients with *de novo* diagnoses of DLBCL treated with curative-intent R-CHOP or similar regimens were included in this study if they met the following criteria: diagnosis between January 1, 2016, and December 31, 2021, at Colsanitas clinics in Colombia, and availability of a complete blood count prior to treatment initiation.

### Exclusion criteria

Patients with previous diagnoses of rheumatologically diseases, cancer, or HIV were excluded as were all patients under treatment with chemotherapy or steroids before the first blood test.

### Variables and data collection

The hematology department database was reviewed and patients who met the inclusion criteria were selected. The follow-up began at the time of the diagnosis and continued until the last available follow-up appointment in the electronic medical record system. All patients were monitored throughout this period

Electronic medical records of patients with DLBCL were reviewed collecting basic patient information, including age at diagnosis, sex, baseline lactic dehydrogenase (LDH) level, Eastern Cooperative Oncology Group performance status (ECOG), Ann Arbor stage, IPI, Revised International Prognostic Index (R-IPI), National Comprehensive Cancer Network-IPI (NCCN-IPI), relapse, and death. The Lugano response criteria were used to assess treatment response [24].

For the proposed analysis, the NLR for each patient was calculated at the time of diagnosis, prior to the initiation of any treatment, including chemotherapy or steroids. The Receiver Operating Characteristic (ROC) curve analysis was performed to determine the optimal NLR cut-off value for predicting the two-year PFS. Subsequently, patients were divided into two groups, patients with higher levels of NLR were included in Group One and those with lower levels in Group Two.

### Outcomes

The main outcome was PFS, defined as the time in months elapsed from diagnosis until disease progression or death from any cause.

The secondary outcome was OS defined as the time elapsed from the date of diagnosis to death from any cause. Both PFS and OS were measured for the same period.

The institutional ethics committee approved this study.

### Statistical analysis

The sample size was calculated with expected sensitivity and specificity of 51.2 % and 79.9 %, respectively using a confidence interval of 95 % and power of 80 %, resulting in a minimum sample size of 122 patients.

Once the data were collected, a mathematical formula was used dividing the absolute neutrophil count by the absolute lymphocyte count for each patient. The ROC curve was constructed using the NLR and the outcome of interest (two-year PFS) to calculate sensitivity, specificity, negative and positive predictive values, and the optimal cutoff point to discriminate the population. The sample was then divided accordingly for analysis. Because the primary outcome was PFS, a time-dependent event, the time-dependent ROC curve was also calculated which showed cumulative sensitivity and dynamic specificity for the performance evaluation of the NLR.

Categorical variables were compared using the chi-square test or Fisher’s exact test, as appropriate, while continuous variables were compared using the Student’s *t*-test or the Wilcoxon rank-sum test. The non-parametric Kaplan-Meier method was used to estimate the OS and DFS and the survival probabilities were compared using the log-rank test.

Proportional hazard regression (Cox) was used to evaluate factors that influenced OS and PFS. Bivariate Cox proportional hazards regression analysis was performed to screen for variables associated with prognosis; those yielding a p-value <0.2 were subsequently included in the multivariate model. A p-value of <0.05 was considered statistically significant. R version 4.3.2 was used for data analysis.

## Results

**Figure 1:**
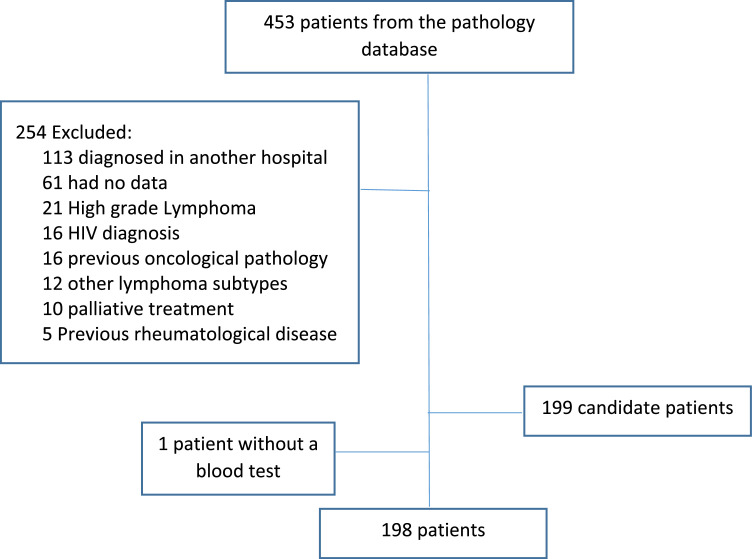
CONSORT flowchart of patients included in the study.

Between 2016 and 2021, 453 patients who were diagnosed with DLBCL were identified; 254 patients were excluded from the study leaving 198 patients (Figure 1). The median follow-up was 45 months and main characteristics of the entire group are listed in Table 1.

Most patients did not have their initial diagnosis at the Colsanitas clinic, so it was not possible to determine if they had received steroid therapy before the blood test.

**Table 1:** Main characteristics of the entire group of Diffuse Large B-Cell Lymphoma patients at diagnosis.

**Variable**	***n*=185**
**Age** – median (interquartile range)	61 (51–72)
**Sex** – n (%)	
Female	89 (48)
Male	86 (52)
**Ann Arbor Staging** – n (%)	
I	12 (6.5)
II	34 (18)
III	32 (17)
IV	107 (58)
**Extranodal involvement** – n (%)	
No	64 (35)
Yes	120 (65)
**International Prognostic Index (IPI)** – n (%)	
Low	50 (27)
Intermediate - low	66 (36)
Intermediate - high	50 (27)
High	18 (9.8)
Unknown	1
**R-IPI** – n (%)	
Very Good	5 (2.7)
Good	111 (61)
Poor	66 (36)
Unknown	3
**NCCN-IPI** – n (%)	
Low	20 (11)
Intermediate - low	85 (47)
Intermediate - high	61 (34)
High	15 (8.3)
Unknown	4

IPI: International Prognostic Index; R-IPI: Revised International Prognostic Index; NCCN-IPI: National Comprehensive Cancer Network-International Prognostic Index.

### Operational characteristics of the neutrophil-to-lymphocyte ratio

The median absolute neutrophil count at diagnosis was 5020 (interquartile range: 3648–6740) and the median absolute lymphocyte count was 1330 (interquartile range: 860–1855).

**Figure 2:**
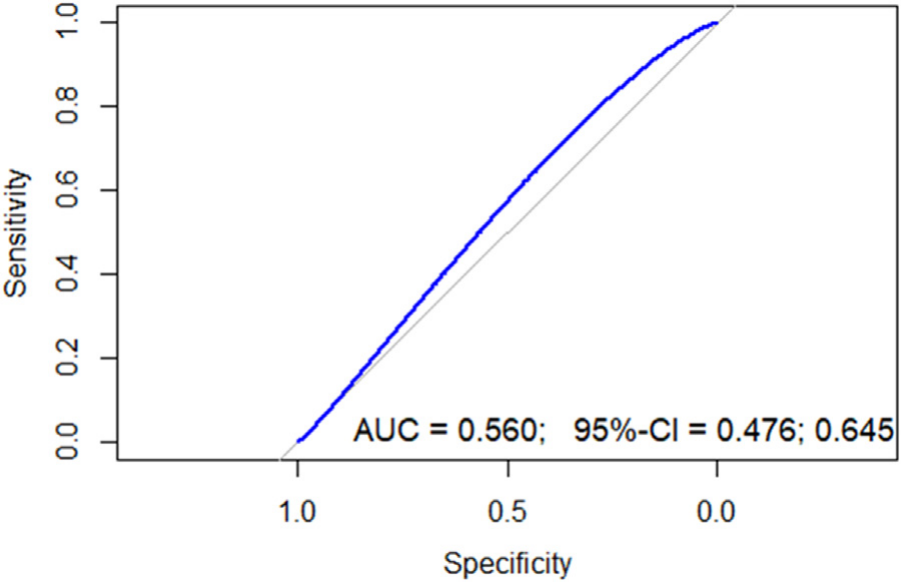
Receiver operating characteristic curve of neutrophil-to-lymphocyte ratio for 24-months progression free-survival.

A ROC curve (Figure 2) showed an area under the curve (AUC) of 0.56 (95 % confidence interval [95 % CI]: 0.47–0.64) with a cutoff point of 3.3: the sensitivity was 66.6 %, specificity 47.8 %, positive predictive value was 48.3 %, and negative predictive value was 65.1 % (Table 2).

AUC: Area under the curve; 95 % CI: 95 % confidence interval.

**Table 2:** Operative characteristics of Neutrophil-to-lymphocyte ratio (NLR) at different cutoff points.

**NLR**	**Sensitivity (%)**	**Specificity (%)**	**LR +**	**LR -**	**PPV (%)**	**NPV (%)**
3.3	70.5	47.8	1.35	0.61	44.4	73.3
3.5	64.7	52.1	1.35	0.67	44.4	71.4
4	54.4	59.1	1.33	0.77	44.1	68.6
4.5	48.5	66.1	1.43	0.77	45.8	68.4
5	47.1	70.4	1.59	0.75	48.5	69.2
5.5	45.6	73.9	1.74	0.73	50.8	69.7
6	39.7	78.2	1.82	0.77	51.9	68.7
**6.2**	**35.3**	**81.7**	**1.93**	**0.79**	**53.3**	**68.1**
6.5	25.0 %	82.6 %	1.43	0.91	45.9 %	65.0 %
7	22.1 %	86.0 %	1.58	0.90	48.3 %	65.1 %
7.5	20.6 %	88.6 %	1.82	0.89	51.8 %	65.3 %

LR+: Likelihood ratio positive; LR–: Likelihood ratio negative; PPV: Positive predictive value; NPV: Negative predicted value; NLR: Neutrophil-to-lymphocyte ratio.

Because of the poor performance of the AUC with a cutoff of 3.3, a cutoff point in the 75th percentile was chosen to improve specificity. Any patient with an NLR >6.2 was considered to have a high NLR: the sensitivity was 35.3 %, specificity was 81.7 %, negative predictive value was 68.1 %, and positive predictive value was 53.8 %. Following the determination of the optimal cut-off value, the cohort was divided into two prognostic groups based on the NLR: Group 1, comprising 45 patients with a high NLR, and Group 2, comprising the remaining 139 patients (Table 3).

**Table 3:** Main characteristics of the patients according Neutrophil-to-lymphocyte ratio (NLR).

**Variable**	**n**	**NLR low (<6.2)**	**NLR High (>6.2)**	**p-value**^2^
		*n* = 139	*n* = 45	
**Age -** Median (interquartile range)	184	61 (52–72)	63 (44–73)	0.7
**Sex** – n (%)	184			0.9
Female		67 (48)	21 (47)	
Male		72 (52)	24 (53)	
**Ann Arbor Staging** – n (%)	184			>0.9
I		10 (7.2)	2 (4.4)	
II		26 (19)	8 (18)	
III		23 (17)	9 (20)	
IV		80 (58)	26 (58)	
**Extranodal involvement** – n (%)	183			0.5
No		50 (36)	14 (31)	
Si		88 (64)	31 (69)	
**IPI** – n (%)	184			0.018
Low		44 (32)	6 (13)	
Intermediate - low		51 (37)	15 (33)	
Intermediate - high		34 (24)	16 (36)	
High		10 (7.2)	8 (18)	
**R-IPI** – n (%)	181			0.050
Very good		5 (3.6)	0 (0)	
Good		89 (65)	22 (50)	
Poor		43 (31)	22 (50)	
**NCCN-IPI** – n (%)	181			0.006
Low		20 (15)	0 (0)	
Intermediate - low		66 (48)	19 (43)	
Intermediate - high		42 (31)	19 (43)	
High		9 (6.6)	6 (14)	

IPI: International Prognostic Index; R-IPI: Revised International Prognostic Index; NCCN-IPI: National Comprehensive Cancer Network-International Prognostic Index.

2 Wilcoxon rank sum test; Fisher’s exact test; Pearson’s Chi-squared test.

### Treatment response

At the end of treatment, complete response was achieved in 104 patients (67.9 %) in Group 2 versus 23 patients (51.1 %) in Group 1. The rates for other responses were as follows: partial response (Group 2: 15.4 %; Group 1: 15.1 %), stable disease (Group 2: 4.6 %; Group 1: 7.6 %), and progression (Group 2: 7.5 %; Group 1: 16.9 %). The difference in overall response rates between the groups was statistically significant (p-value = 0.035)

### Survival analysis

#### Progression-free survival

**Figure 3:**
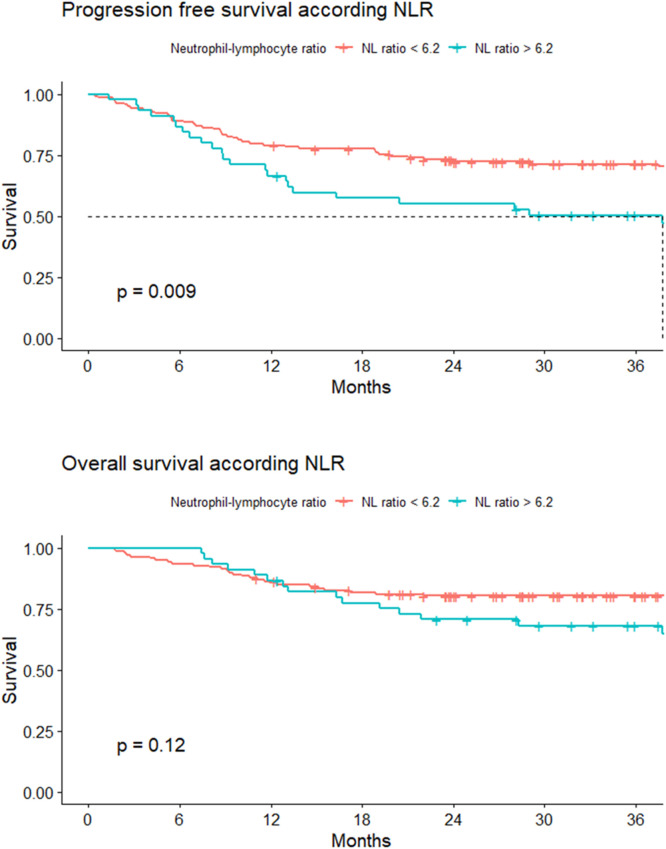
Progression-free survival (PFS) and overall survival (OS) according to neutrophil-to-lymphocyte ratio.

The 24-month PFS for Group 1 was 55.2 %; (95 % CI: 42.3–71.9 %), and for Group 2, it was 73.2 % (95 % CI: 66.2–81 %). The hazard ratio (HR) for Group 2 was 0.62 (95 % CI: 0.44–0.89, p-value = 0.009 - Figure 3).

#### Overall survival

For both groups, the median OS was not reached. The 24-month OS was 70.7 % (95 % CI 58.5 −85.5 %) and 80.4 % (95 % CI: 66.2–87.3 %) for Groups 1 and 2, respectively. A HR of death of 0.71 (95 % CI: 0.45–1.1) was estimated for Group 2 (Figure 3).

### Multivariate analysis

Initially, bivariate Cox proportional hazards regression analysis was performed to identify variables for inclusion; those with a p-value <0.2 were subsequently entered into the multivariate model.

NLR, age over 60 years, ECOG ≥2, involvement of more than two extranodal sites, bone marrow involvement, and bulky mass were chosen to construct the multivariate model. The NLR continued to have a relationship with PFS after adjustment in the multivariate model (Table 4).

**Table 4:** Univariate and multivariate Analysis - Cox model for progression-free survival.

Analyzed variables	**Univariate analysis**		**Multivariate analysis**	
	**HR (95% CI)**	**p-value**	**HR (95% CI)**	**p-value**
NLR ≥6.2	1.58 (1.11–2.26)	0.009	1.65 (1.15–2.35)	0.006
Age >60 years	1.024 (1.008–1.042)	0.004	1.03 (1.01–1.04)	0.002
Non-germinal center	1.01 (0.57–1.77)	0.97		
ECOG ≥2	1.97 (0.84–4.59)	0.11		
Stage IV Ann Arbor	0.81 (0.32–2.08)	0.67		
Extranodal involvement	1.03 (0.62–1.72)	0.89		
≥2 Extranodal sites	1.65 (1.01–2.74)	0.049		
Bone marrow involvement	1.98(0.98–4.01)	0.05	2.26 (1.11- 4.63)	0.025
Bulky mass	1.45 (0.89–2.34)	0.12	1.87(1.13–3.08)	0.014

HR: Hazard ratio; 95 % CI: 95 % confidence interval; NLR: Neutrophil-to-lymphocyte ratio; ECOG: Eastern Cooperative Oncology Group performance status.

## Discussion

In this study, the standard ROC curve of the NLR yielded an AUC of 0.56 with a cutoff point of 3.3; this was considered unsatisfactory. Consequently, the decision was made to select a cutoff point at the 75th percentile giving a higher specificity. The cutoff point for NLR was determined to be 6.2 with 35.3 % sensitivity and 81.7 % specificity.

There were statistically significant differences in 24-month PFS (p-value = 0.009). However, this study did not demonstrate significant differences in 24-month OS.

Other studies showed poor performance for the NLR in the ROC curve analysis. In 2021, Hasan published an analysis of 136 DLBCL patients who were divided into low and high NLR groups using a cutoff value of 2.8. The AUC for NLR was 0.512 (95 % CI: 0.41–0.61) with 57.4 % sensitivity and 55.1 % specificity (p-value = 0.81), but did not show differences in the five-year PFS (61.8 %; 95 % CI: 47.3–73.4 % versus 58.5 %; 95 % CI: 45.3–72.4 %; p-value = 0.41) or OS (63.5 %; 95 % CI: 50.4–76.9 % versus 56 %; 95 % CI: 43–69 %; p-value = 0.42) [25].

According to the ROC analysis of the Latin American Group of Lymphoproliferative Diseases (GELL), the optimal cutoff point is 4 with sensitivity and specificity of 50 % and 60 %, respectively and an AUC of 0.59. Significant differences were found between the groups for the 5-year OS (75 %; 95 % CI: 68 %−81 % versus 48 %; 95 % CI: 35 %−60 %, respectively; HR: 2.09; 95 % CI:1.43–3.30; p-value <0.001) [8].

According to meta-analyses data of nine studies with 1984 patients, NLR was a predicted factor for PFS (HR: 1.64; 95 % CI: 1.36–1.98; I^2^ = 36.9 %). Two of these studies did not demonstrate significant differences [21,26]. The reported cutoff point in all studies was highly variable with values between 2.32 to 4.35 and the absolute value of the relationship was not associated with survival [16].

The meta-analyses data showed a NLR as a prognostic factor of OS (HR: 1.84; 95 % CI: 1.52–22; I^2^ = 7.3 %) [27]. Two of these studies did not show differences in OS: Melchardt et al. and Ho et al. [28,29].

The limitations associated with this study include its retrospective design, being conducted in a single medical center, and obtaining a ROC curve with a low AUC. However, this is the only known study that evaluated an easily accessible biomarker for any hospital in a large sample of patients in the Colombian population. The collected patient data were sufficient to identify significant differences according to the previously calculated sample size, and the data quality was satisfactory. There were low losses during follow-up, and the methodology used, conducting a standard ROC curve analysis, was appropriate for a time-to-event outcome.

In conclusion, in this study, the NLR with a cutoff point of 6.2 was found to be a significant prognostic marker for 24-month PFS but not for OS. After multivariate analysis, the NLR remained a prognostic variable for PFS, suggesting that it could be used as a complementary tool of daily prognostic scores in clinical practice.

## Conflicts of interest

There are no conflicts of interest to report.
